# Kinematic Synergy of Multi-DoF Movement in Upper Limb and Its Application for Rehabilitation Exoskeleton Motion Planning

**DOI:** 10.3389/fnbot.2019.00099

**Published:** 2019-11-29

**Authors:** Shangjie Tang, Lin Chen, Michele Barsotti, Lintao Hu, Yongqiang Li, Xiaoying Wu, Long Bai, Antonio Frisoli, Wensheng Hou

**Affiliations:** ^1^Key Laboratory of Biorheological Science and Technology, Ministry of Education, Bioengineering College, Chongqing University, Chongqing, China; ^2^Chongqing Key Laboratory of Artificial Intelligence and Service Robot Control Technology, Chongqing, China; ^3^PERCRO Laboratory, TeCIP Institute, Scuola Superiore Sant'Anna, Pisa, Italy; ^4^Chongqing Engineering Research Center of Medical Electronics Technology, Chongqing, China; ^5^College of Mechanical Engineering, Chongqing University, Chongqing, China

**Keywords:** kinematic synergies, upper limb movements, principal component analysis, motion planning, inter-joint coordination

## Abstract

It is important for rehabilitation exoskeletons to move with a spatiotemporal motion patterns that well match the upper-limb joint kinematic characteristics. However, few efforts have been made to manipulate the motion control based on human kinematic synergies. This work analyzed the spatiotemporal kinematic synergies of right arm reaching movement and investigated their potential usage in upper limb assistive exoskeleton motion planning. Ten right-handed subjects were asked to reach 10 target button locations placed on a cardboard in front. The kinematic data of right arm were tracked by a motion capture system. Angular velocities over time for shoulder flexion/extension, shoulder abduction/adduction, shoulder internal/external rotation, and elbow flexion/extension were computed. Principal component analysis (PCA) was used to derive kinematic synergies from the reaching task for each subject. We found that the first four synergies can explain more than 94% of the variance. Moreover, the joint coordination patterns were dynamically regulated over time as the number of kinematic synergy (PC) increased. The synergies with different order played different roles in reaching movement. Our results indicated that the low-order synergies represented the overall trend of motion patterns, while the high-order synergies described the fine motions at specific moving phases. A 4-DoF upper limb assistive exoskeleton was modeled in SolidWorks to simulate assistive exoskeleton movement pattern based on kinematic synergy. An exoskeleton Denavit-Hartenberg (D-H) model was established to estimate the exoskeleton moving pattern in reaching tasks. The results further confirmed that kinematic synergies could be used for exoskeleton motion planning, and different principal components contributed to the motion trajectory and end-point accuracy to some extent. The findings of this study may provide novel but simplified strategies for the development of rehabilitation and assistive robotic systems approximating the motion pattern of natural upper-limb motor function.

## Introduction

Hemiplegia, or unilateral paresis, is currently reported to be one major cause of physical disability in the middle-aged and elderly (Paul et al., 2002; Gert et al., 2003). Patients often experience autonomic difficulties in daily life due to the unilateral motor dysfunction. Recent works on upper-limb robotic training experiments exhibited substantial improvements on joint motions in either the horizontal plane or three-dimensional space for the patients of motor impairment with wearable exoskeletons (Kwakkel et al., 2008; Jarrassé et al., 2014). Therefore, exoskeleton technology has attracted extensive attentions for its noteworthy value of assisting multi-joint rehabilitation movement and daily life performance (such as reaching and grasping) (Frisoli et al., 2012). Pilot studies suggested that spatiotemporal motion patterns that well match the nature of upper-limb joint movement plays a key role in the long lasting effects in rehabilitation (Liu et al., 2018). A typical upper limb movement is fulfilled with spatiotemporal motor coordination of multiple joints, and kinematic synergy among limb joints have been widely explored as a control principle for motor function (Tomita et al., 2017). However, little is known how to effectively use kinematic synergies of upper limb movement in exoskeleton motion planning for motor assistive purpose.

Numerous robotic devices for upper limb rehabilitation have been developed with kinematic patterns mimicking actual human arm movements with multiple degree of freedoms (DoF). The kinematics of multi-joint coordination were extracted and implemented in exoskeleton to conduct activities of daily life (ADLs). Johnson et al. (2001) designed a 5-DoFs motorized assistive device with a 3-DoFs cable driven shoulder structure to enable patients with arm disorders to perform controlled movements, strengthening exercises, and continuous passive motion according to selective modes. Xu and Qiu (2013) proposed an alternative design of a flexible continuum exoskeleton apparatus to collaborate with human movements for the shoulder joint only. Most of these devices were programmed to certain trajectories for rehabilitation training without full considerations of actual continuous multi-joint movement modes, and may obstruct smooth performance of consecutive upper limb movements (Ding et al., 2014). To overcome the kinematic redundancy in reaching movements, Li et al. introduced an exponential method of human motor control strategy, which demonstrated accuracy improvements for real-time motion control of designed upper limb exoskeleton (Li et al., 2015). However, the regulation strategies in central nervous system (CNS) for multi-joint coordination in upper limb tasks were correlated to complex movement patterns (Scano et al., 2017). A simulated framework based on muscular and postural synergy indicated that a robotic arm with multi-DoF could generate arbitrary trajectories similar to human natural movements for the end-effector (Liu and Xiong, 2013), but also increased redundancy that deteriorated the motion control. Several studies attempted to simplify the motor control of assistive device for upper limb actuating planar or three-dimensional motion performance, such as the impedance control (Hogan et al., 1992) and mirror-image movement enabler technique (Lum et al., 2004).

The temporal and spatial inter-limb coordination plays a key role in upper limb rehabilitation. The surface electromyography (sEMG) signal was frequently employed to control multi-DoF mechanical arm (Hang et al., 2014), however, the sEMG signal did not directly reflect those complex temporal multi-joint coordination across limbs (Merad et al., 2018). Alternative motion kinematics model has also derived via fitting a high-order polynomials based on sEMG analysis to estimate the multi-joint angles of upper limb movements (Ding et al., 2014). However, sEMG-based muscle activities were generally represented in high dimensional feature space. It was difficult to meet the robustness requirement of complicated multi-DOF arm motions (Ding et al., 2016). Other strategies have also been developed to optimize the near-natural trajectories of assistive equipment. For example, a mathematical model based on a criterion function to characterize planar two-joint arm movements was formulated by Flash and Hogan, and the predicted trajectories matched experimental observations of real human performance well in a horizontal plane (Flash and Hogan, 1987). Nevertheless, how to temporally represent motion patterns of the multi-joint coordination similar to actual trajectory of human arm movements still left an open question.

**Figure 1:**
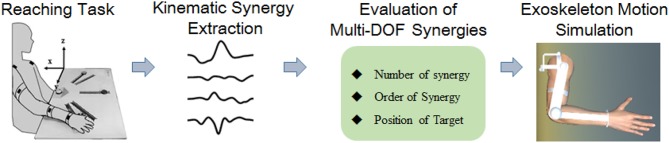
Block diagram of this work.

Kinematic synergy based on classical neuromechanic theories (Bernstein, 1967) is another important concept in motor coordination. Existing evidence suggests that the CNS generates motor command to co-activate multiple muscles working with specific extents, which was generally referred to as “synergy” (Turvey, 2007; Mukta et al., 2015). During motion behaviors, the neural control strategies were modulated to regulate synergistic patterns dynamically to meet the task requirements (Burns et al., 2017). Several pilot studies have employed kinematic synergies to evaluate the inter-joint movement patterns of different human motor approaches (Tresch et al., 2006; Chen and Xiong, 2013). Artificial neural network (Devi et al., 2013), linear discriminant analysis (LDA) (Ramana Vinjamuri et al., 2015), and several other algorithms have been introduced to derive synergies; however, principal component analysis (PCA) is most frequently employed for kinematic synergies analysis (Ramana et al., 2010; Chen and Xiong, 2013; Patel et al., 2016; Burns et al., 2017). Burns et al. (2017) successfully used PCA method to derive time-varying kinematic synergies of bilateral upper arm reaching movement. Bockemühl et al. (2010) analyzed the kinematics complexity of human catching movement, and found that the first three principal components captured more than 97% of variance. Further study confirmed that human upper limb trajectories can be reconstructed by a linear combination of few principal time-dependent functions (Averta et al., 2017). Also, researchers employed synergy analysis as inputs to control the rehabilitation mechanical system, such as upper limb exoskeleton rehabilitation robot (Liu and Xiong, 2013) or dexterous hand for grasping movements (Catalano et al., 2014). However, few efforts have been made to manipulate the motion control based on human kinematic synergies. This work analyzed the spatiotemporal kinematic synergies of right arm reaching movement and investigated their potential usage in upper limb assistive exoskeleton motion planning (see Figure 1). Therefore, we employed PCA algorithm to analyze the spatiotemporal kinematic synergistic pattern of the shoulder and elbow joint in reaching activities and simulated the motion planning for upper limb assistive exoskeleton with kinematic synergies.

## Subjects and Methods

This study was conducted at the Perceptual Robotics Laboratory (Pisa, Italy). Ten healthy subjects (eight males and two females, with the age of 26 ± 2) participated in the study after informed consent was obtained. All subjects were right handed and confirmed free of any arm neuromuscular disorder or previous joint injury. The experimental procedures were conducted in accordance with the Declaration of Helsinki and approved by the Ethical Review Board of Scuola Superiore Sant'Anna.

### Data Acquisition

**Figure 2:**
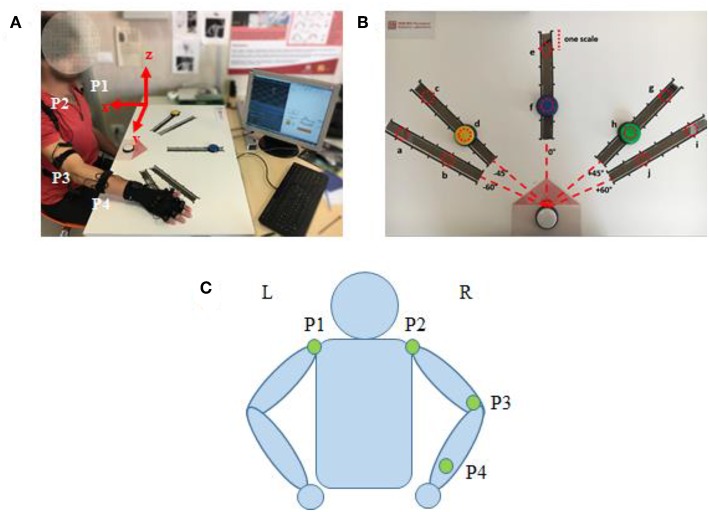
**(A)** Subject interacting with the Perception Neuron device (incorporated with IMU), sitting at the desk while wearing the wearable system and performing tasks. **(B)** The cardboard used in the experiment: the red dotted circles represent the 10 positions where the targets can be placed (named from “a” to “j”). **(C)** Kinematic model with specific locations of the Perception Neuron sensors. The green circle indicates the sensor (IMU) in the corresponding position has been activated.

An IMU-based motion capture system (Perception Neuron, Noitom Technology Ltd., Beijing, China) was used to record motion data of the subjects' upper limb during tasks. Four IMU sensors were placed on the subjects, as shown in Figure 2C (P1–P4): left shoulder, right shoulder, right elbow, right wrist. Then, the spatial position of shoulder, elbow, wrist, and their trajectory of upper limb motion could be recorded during test. Referred as an experimental report (Burns et al., 2017), frame calibrations were performed before the data acquisition as: (1) positive x extended backward away from the subject; (2) positive y extended to the right side of subject, and (3) positive z extended upwards toward the ceiling. The axis x, y, and z were illustrated in Figure 2A. Therefore, the data collected by this motion capture system can scale the right arm motion pattern with respect to subject's torso. The sampling frequency is 120 samples per second.

### Experimental Procedure

The participants were asked to reach the target button placed on a cardboard in front of them. Three buttons (yellow, blue, and green) were designed as targets and a white button was assigned as the start point. A LED (light-emitting diode) was embedded in white button to cue a reaching task beginning, whereas a LED was embedded in yellow, blue or green button to show which target should be reached in a trial. Five linear tracks in different directions were defined by the angles from the central track (Figures 2B,C) as: −60, −45, 0, 45, and 60°. The central track (i.e., 0°) was directly in front of the subject. The target buttons were placed on the selected 3 tracks. We assigned the yellow button to one of the contralateral two tracks (−60 or −45°), blue button to the central track (0°), and green button to one of the ipsilateral two tracks (45 or 60°). On each track, 6 different locations with 5 cm interval were designed for the placement of target buttons, however, only 2 locations were used (the second and the fifth scales) in the experiments. Therefore, a total of 10 possible target locations, named in alphabetical order as “a” to “j,” were involved (Figure 2B). The distance between the start and the target position was designed as about 80% of the maximum distance that the participant can reach.

The reaching task trial was conducted as: (1) pressing the start button (white); (2) pressing a button located at ipsilateral (green), central (blue) or contralateral (yellow) as the reaching task required; (3) returning to the start position (white). An acoustic trigger was used as the start cue for participants to reach and press the target button. Each target was pressed six times, but the target order was randomly assigned. The instructions for reaching movements were given with a randomized interval between 1 and 3 s. Each of these ten Participants were required to avoid any redundant movement during the whole procedure of task performance. A more detailed experimental procedure was described in a previous report (Tang et al., 2018).

### Kinematics Measurement of Right Upper Limb

During our reaching task, the upper limb and joints performed spatial motions, which could be projected to the calibrated frame of axes. Then, the motion pattern of limb and joint can be evaluated through calculating the angular course at each time point. As illustrated in Figure 2C, the kinematics chain of arm was modeled with four segments passing through the five aforementioned joints to evaluate the following angles: shoulder flexion/extension (SFE), shoulder abduction/adduction (SAA), shoulder internal/external rotation (SIR), and elbow flexion/extension (EFE). The kinematics is defined by the following vectors:

(1) $$\begin{array}{r} p_{i}=\left[x_{i},y_{i},z_{i}\right] \end{array}$$

(2) $$\begin{array}{r} {\bar{s}}_{12}=p_{1}-p_{2} \end{array}$$

(3) $$\begin{array}{r} {\bar{s}}_{32}=p_{3}-p_{2} \end{array}$$

(4) $$\begin{array}{r} {\bar{s}}_{43}=p_{4}-p_{3} \end{array}$$

where ${\bar{s}}_{n}\;$ is a normal vector to the sagittal plane, and *p*_*i*_(*x*_*i*_, *y*_*i*_, *z*_*i*_) is the spatial coordinates of each joint. The angle of SFE, SAA, SIR, and EFE are then calculated as θ_*sf*_, θ_*sa*_, θ_*si*_, and θ_*ef*_, respectively. ${\bar{s}}_{ij_{x}}=0$ denotes the *x* coordinate in vector ${\bar{s}}_{ij}$ set to 0.

(5) $$\begin{array}{r} \theta_{sf}={\text{cos}}^{-1}\frac{{\bar{s}}_{32}\cdot {\bar{s}}_{n}}{\left\|{\bar{s}}_{32}\|\|{\bar{s}}_{n}\right\|},\;{\bar{s}}_{32_{z}}=0 \end{array}$$

(6) $$\begin{array}{r} \theta_{sa}={\text{cos}}^{-1}\frac{{\bar{s}}_{32}\cdot {\bar{s}}_{n}}{\left\|{\bar{s}}_{32}\|\|{\bar{s}}_{n}\right\|},\;{\bar{s}}_{32_{x}}=0 \end{array}$$

(7) $$\begin{array}{r} \theta_{si}={\text{cos}}^{-1}\frac{{\bar{s}}_{12}\cdot {\bar{s}}_{32}}{\left\|{\bar{s}}_{12}\|\|{\bar{s}}_{32}\right\|},\;{\bar{s}}_{12_{y}}=0,\;{\bar{s}}_{32_{y}}=0 \end{array}$$

(8) $$\begin{array}{r} \theta_{ef}={\text{cos}}^{-1}\frac{{\bar{s}}_{42}\cdot {\bar{s}}_{23}}{\left\|{\bar{s}}_{43}\|\|{\bar{s}}_{23}\right\|} \end{array}$$

### Synergy Derivation

**Figure 3:**
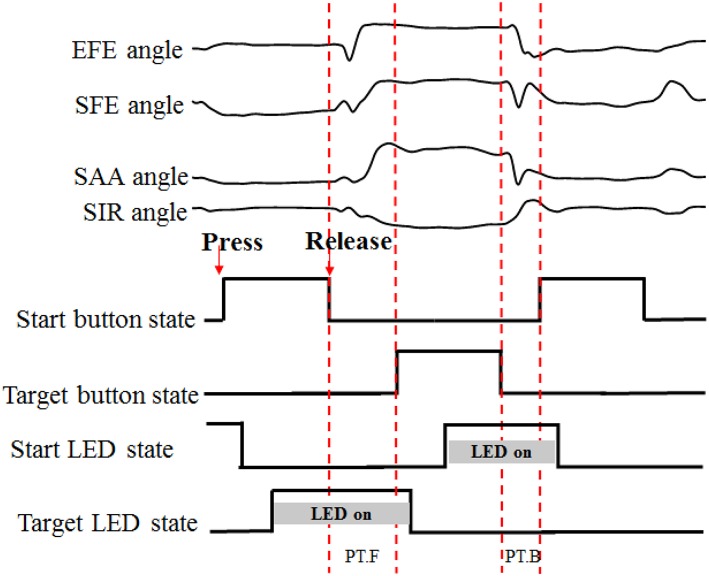
Data segmentation process. “PT” for performance time, “F” and “B” for forward and backward, respectively. When the start button or target button was pressed, the LED was turned off.

The time-markers of action performance (button pressed/released and LED on/off states) were recorded through a Simulink model in MATLAB (the Mathworks, Natich, MA, USA). A complete recording set was segmented between the release and re-press of the white start button (Figure 3). The PT.F (forward-performance time) was used to segment the angular data of forward movement of reaching task trial by trial.

After data segmentation, the angular velocities were calculated by the derivative of the temporal joint angle profiles. For further processing, the angular velocity data of a reaching trial was resampled to 150 samples for each DOF. Then, the *ith* sample of a trial was used to construct a 6 × 4 sub-matrix as following:

(9) $$\begin{array}{r} W\left(i\right)=\left[\begin{array}{cccc} \omega_{1}^{1}\left(i\right) & \omega_{2}^{1}\left(i\right) & \omega_{3}^{1}\left(i\right) & \omega_{4}^{1}\left(i\right)\\ \omega_{1}^{2}\left(i\right) & \omega_{2}^{2}\left(i\right) & \omega_{3}^{2}\left(i\right) & \omega_{4}^{2}\left(i\right)\\ \vdots & \vdots & \vdots & \vdots \\ \omega_{1}^{6}\left(i\right) & \omega_{2}^{6}\left(i\right) & \omega_{3}^{6}\left(i\right) & \omega_{4}^{6}\left(i\right) \end{array}\right]\left(i=1,\;2,\;3,\;\cdots \;,\;150\right) \end{array}$$

Where the subscript (row variable) and superscript (column variable) represent the joint DOFs and task repetitions, respectively. Then, a 6 × 600 matrix *W* composed of all 150 sub-matrixes was constructed to represent the six repetitions of a target reaching task to each subject.

(10) $$\begin{array}{r} W=\left[\begin{array}{cccc} W\left(1\right) & W\left(2\right) & \ldots & W\left(150\right) \end{array}\right] \end{array}$$

Thus, *W* was 6-by-600 matrix, which was then given as input to the PCA algorithm to extract spatiotemporal synergies. Then, singular value decomposition (SVD) algorithm was performed on angular velocity matrix *W* to derive kinematic synergies. Three-component matrices *U*, Σ, and *S* were computed from *W*, as shown in:

(11) $$\begin{array}{r} W=U\Sigma S \end{array}$$

*U* and *S* are orthogonal matrices, and Σ is a diagonal matrix. In this case, *U* is a 6-by-6 matrix, which has orthonormal columns and *S* is a 600-by-600 matrix with orthogonal rows. The diagonal matrix Σ is of 6-by-600 and the diagonal elements of Σ correspond to the singular values (λ_*i*_) of *W*.

(12) $$\begin{array}{r} \Sigma =\left[\begin{array}{ccccccccc} \lambda_{1} & 0 & 0 & 0 & 0 & 0 & 0 & \cdots & 0\\ 0 & \lambda_{2} & 0 & 0 & 0 & 0 & 0 & \cdots & 0\\ 0 & 0 & \lambda_{3} & 0 & 0 & 0 & 0 & \cdots & 0\\ 0 & 0 & 0 & \lambda_{4} & 0 & 0 & 0 & \cdots & 0\\ 0 & 0 & 0 & 0 & \lambda_{5} & 0 & 0 & \cdots & 0\\ 0 & 0 & 0 & 0 & 0 & \lambda_{6} & 0 & \cdots & 0 \end{array}\right] \end{array}$$

*S* matrix is defined as in equation below:

(13) $$\begin{array}{r} S=\left[\begin{array}{ccccccc} s_{1}^{1}\left(1\right) & \cdots & s_{4}^{1}\left(1\right) & \cdots & s_{1}^{1}\left(t_{\text{max}}\right) & \cdots & s_{4}^{1}\left(t_{\text{max}}\right)\\ \vdots & \; & \vdots & \; & \vdots & \; & \vdots \\ s_{1}^{m}\left(1\right) & \cdots & s_{4}^{m}\left(1\right) & \cdots & s_{1}^{m}\left(t_{\text{max}}\right) & \cdots & s_{4}^{m}\left(t_{\text{max}}\right)\\ \vdots & \; & \vdots & \; & \vdots & \; & \vdots \\ s_{1}^{M}\left(1\right) & \cdots & s_{4}^{M}\left(1\right) & \cdots & s_{1}^{M}\left(t_{\text{max}}\right) & \cdots & s_{4}^{M}\left(t_{\text{max}}\right) \end{array}\right] \end{array}$$

The first *m* rows of *S* are called the first *m* principal components, or “synergies” and *M* was the maximal number of synergies. The approximation matrix $\tilde{W}$ can be composed by the first *m* columns of *U*, *m*-by-*m* of Σ (the other values are replaced by zeros), and *m* rows of matrix *S*. The product *U*_*m*_*diag*{λ_1_, λ_2_, ⋯ , λ_*m*_} is called the weight matrix.

(14) $$\begin{array}{r} \tilde{W}=U_{m}diag\left\{\lambda_{1},\lambda_{2},\cdots \;,\lambda_{m}\right\}S_{m} \end{array}$$

The fraction of sum-squared variance can be calculated from the diagonal elements of Σ and this index was denoted as *K*.

(15) $$\begin{array}{r} K=\frac{\lambda_{1}^{2}+\lambda_{2}^{2}+\cdots +\lambda_{k}^{2}}{\lambda_{1}^{2}+\lambda_{2}^{2}+\cdots +\lambda_{M}^{2}}\left(k=1,\;2,\;3,\;\cdots \;,\;M\right) \end{array}$$

*K* was used to determine how many principal components were sufficient to represent the whole data. The index threshold of 94% variance was used to determine the best number of synergies.

The angular velocity signal was reconstructed by selecting different numbers of synergies. The reconstruction error *e* between measured ω_*j*_(*t*) and reconstructed $\tilde{\omega_{j}}\left(t\right)$ across joints (*J* = 4) was computed for each reaching movement. One-way analysis of variance (ANOVA) was performed based on normalized reconstruction error according to different number of recruited synergies.

(16) $$\begin{array}{r} e=\frac{\sum_{j=1}^{J}\sum_{0}^{t}{\left(\omega_{j}(t)-{\tilde{\omega }}_{j}(t)\right)}^{2}}{\sum_{j=1}^{J}\sum_{0}^{t}\omega_{j}{\left(t\right)}^{2}} \end{array}$$

### Motion Planning for Upper Limb Assistive Exoskeleton

**Figure 4:**
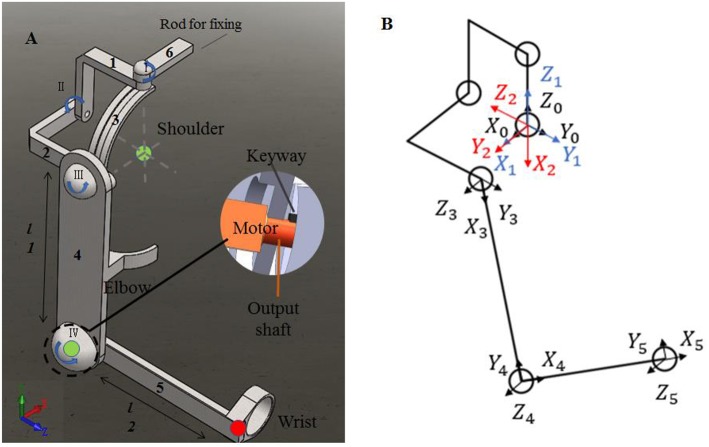
The assistive exoskeleton model and its coordinate system. **(A)** Assistive exoskeleton model for right upper limb assistive exoskeleton model; **(B)** the coordinate system of assistive exoskeleton for Denavit-Hartenberg (D-H) model. The red circle dot in left panel was the endpoint position in D-H model during reaching movement (the end point of bracket 5).

To evaluate the impact of kinematic synergy on arm motion control, an upper limb rehabilitation exoskeleton model was built in SolidWorks (Dassault Systemes, Massachusetts, USA). The simulated exoskeleton was used to guide the joints of right arm moving in 3-DoFs rotation for shoulder joint and 1-DoF flexion/extension for elbow joint. As illustrated in Figure 4, the exoskeleton was driven by four stepping motors within the controlled range of motion (ROM). The ROMs of EFE, SFE, SAA, and SIR joint for reaching movements were set as 0–135, 0–100, 0–90, and 0–105°, respectively. Motor I was assigned to drive the device rotating horizontally to achieve internal/external rotations of the shoulder joint. Motor II could enable the rotation and slide of bracket 2 on the chute 3 to perform the shoulder abduction/adduction. Motor III was used to control arm bracket 4 rotating around the bracket 2 to execute the flexion/extension. Motor IV rotated the forearm bracket 5 around the upper-arm bracket 4 to perform flexion/extension of the elbow joint. The description of motors in this model were detailed in Table 1.

**Table 1:** Description of motors used in model.

**Degree of freedoms (DoF)**	**The range of motion (ROM)**	**Driven motor**	**Rotation mode**	**Cooperate with brackets**
SIR	0–105°	I	Rotating in transverse plane	Rotate horizontally around the bracket 1
SAA	0–90°	II	Rotating in coronal plane	Rotate and slide of bracket 2 on the chute 3
SFE	0–100°	III	Rotating in sagittal plane	Control bracket 4 rotating around bracket 2
EFE	0–135°	IV	Rotating around bracket 4	Control bracket 5 rotating around bracket 4

*SIR was the arm rotating in transverse plane, and the upper arm moving to right side of sagittal plane was set as positive; SAA was the arm rotating in coronal plane, and the upper arm moving outward was set as positive; SFE was the arm rotating in sagittal plane, and the upper arm moving forward was set as positive*.

In order to evaluate the effect of kinematic synergy on arm movement control, an exoskeleton Denavit-Hartenberg (D-H) model was established. This model consisted of five coordinate systems (see Figure 4B). The base coordinate system {0} was set at the shoulder joint center, whereas the coordinate system {1}, {2}, {3}, and {4} were specified with SIR, SAA, SFE, and EFE, respectively. In the Denavit-Hartenberg (D-H) model, the origins of coordinate system {0}, {1}, and {2} were located at the center of the shoulder joint; the origin of coordinate system {3} was located at the position of motor for flexion and extension, and the origin of coordinate system {4} was located at the motor position of elbow joint. The origin of coordinate system {5} was located at the position of the end of the exoskeleton (see the red circle dot in Figure 4A).

To quantify the effect of kinematic synergy on reaching movement control, we calculated the spatial position of the end-point of the exoskeleton model when angular variables was applied to D-H matrix. The Denavit-Hartenberg (D-H) matrix for our exoskeleton model was specified as

(17) $$\begin{array}{r} \left[\begin{array}{cccc} \cos\theta_{i} & -\sin\theta_{i} & 0 & a_{\left(i-1\right)}\\ \sin\theta_{i}\cos\alpha_{\left(i-1\right)} & \cos\theta_{i}\cos\alpha_{\left(i-1\right)} & -\sin\alpha_{\left(i-1\right)} & -\sin\alpha_{\left(i-1\right)}d_{i}\\ \sin\theta_{i}\sin\alpha_{\left(i-1\right)} & \cos\theta_{i}\sin\alpha_{\left(i-1\right)} & \cos\alpha_{\left(i-1\right)} & \cos\alpha_{\left(i-1\right)}d_{i}\\ 0 & 0 & 0 & 1 \end{array}\right] \end{array}$$

(18) T05=T0112T23T34T45T

Where *a*_*i*−1_ was the distance between *Z*_*i*−1_ to *Z*_*i*_ along *X*_*i*−1_, α_*i*−1_ was the angle at which *Z*_*i*−1_ to *Z*_*i*_ rotated around *X*_*i*−1_, *d*_*i*_ was the distance measured from *X*_*i*−1_ to *X*_*i*_ along *Z*_*i*_, θ_*i*_ was the angle at which *X*_*i*−1_ to *X*_*i*_ rotated around *Z*_*i*_. The Denavit-Hartenberg (D-H) parameters were listed in Table 2.

**Table 2:** Denavit-Hartenberg (D-H) parameter.

***i***	***a*_(i - 1)_**	**α_(i - 1)_**	***d*_i_**	**θ_i_**
1	0	0	0	θ_*si*_
2	0	90	0	θ_*sa*_ − 90
3	*r*∗sinθ_*sa*_	−90	*r*	θ_*sf*_
4	*l*_1_	0	0	180 − θ_*ef*_
5	*l*_2_	0	0	0

*In our model, r = 70 mm, the radius of the exoskeleton slip ring; l_1_ = 240 mm, the length upper arm in exoskeleton; l_2_ = 225 mm, the length of forearm in exoskeleton; θ_si_, θ_sa_, θ_sf_, θ_ef_ represented angles of SIR, SAA, SFE, EFE, respectively*.

## Results

### Synergy Extraction Using PCA

**Figure 5:**
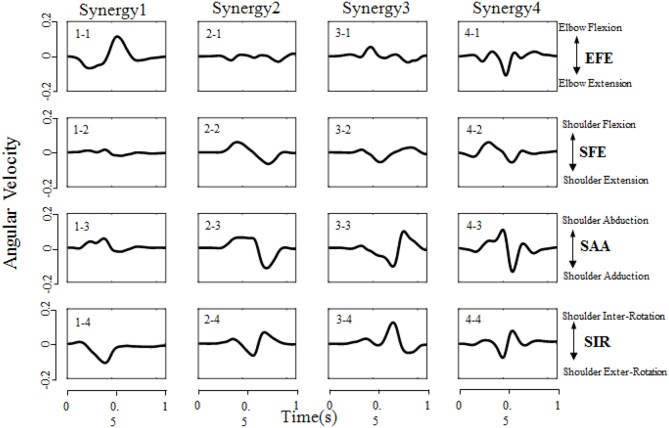
Angular velocity profile based on the first four synergies of subject 9 performing the task of reaching target “e” (target location was illustrated in Figure 2B). Each column corresponds to a specific synergy which have a duration time of 1 second (x-axis), and each row corresponds to a DoF (EFE, SFE, SAA, and SIR, respectively). The Positive/Negative angular velocity amplitudes (y-axis) indicates the movements of elbow flexion/extension (EFE), shoulder flexion/extension (SFE), shoulder abduction/adduction (SAA), and shoulder internal/external rotation (SIR), respectively.

All designed experiments have been successfully repeated 6 times at each target location in randomized order, and the kinematic data of right upper limb are collected during reaching task being conducted. Spatiotemporal kinematic synergies are derived using PCA subject by subject. Figure 5 presents an example of angular velocity profile based on the first four synergies of subject 9 performing the task for reaching target “e.” Here, the first synergy (column 1) involving shoulder flexion (SFE), abduction (SAA) and external rotation (SIR) clearly demonstrate a forward reaching movement. In the same time, an elbow extension (EFE) was conducted and guide his/her right arm moving to press a target. Synergy 2 (column 2) shows a similar shoulder kinematic trend, with higher amplitudes and longer duration time for the shoulder abduction (SAA) and flexion (SFE). Synergy 3 (column 3) consists of a shoulder extension (SFE), a combination of a shoulder adduction followed by an abduction (SAA), and a combination of internal rotation followed by a slight external rotation (SIR). The corresponding elbow angular velocity profile (EFE) illustrates the procedure of “flexion-extension-flexion.” Synergy 4 (column 4) tends to behave similarly as synergy 2 (column 2), except that the elbow movement exhibits repeated flexion/extension with a shorter execution time (EFE).

**Figure 6:**
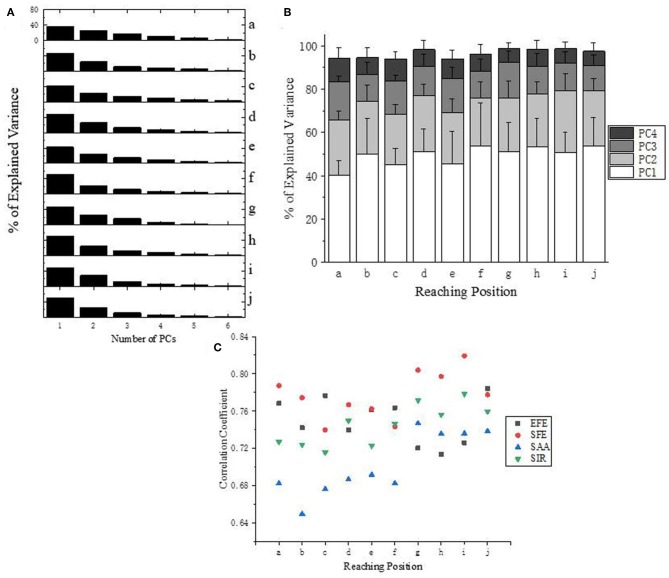
**(A)** Explained Variance for all target positions (a–j) of subject 10. **(B)** Explained Variance of first four PCs at all reaching position across all subjects. **(C)** Correlation analysis of the first PC (synergy 1) across subjects according to different DoFs.

Figure 6A presents the explained variance of PCs (or “synergies”). As shown in Figure 6B, four PCs are sufficient to capture more than 94% of the data's variance for all target positions (a-j). Moreover, the first three PCs account for at least 87% of the variance, and the first two PCs are also able to explained at least 64% of the variance. Specifically, the first two PCs at position “f” accounting for variance even achieve 78%.

Correlation analysis of the first PC (synergy 1) has been carried out for each DoF of upper limb across subjects (Figure 6C). Ipsilateral reaching tasks (target position at g–j) shows higher correlations than the central and contralateral tasks (a–f), which suggests that personalized kinematic parameters vary less in tasks of reaching the closer or more skilled positions. The high level of coefficient value for SFE (red dots) also reveals that the shoulder flexion/extension exhibits better stability across subjects than the other three DoFs (EFE, SAA, and SIR).

**Figure 7:**
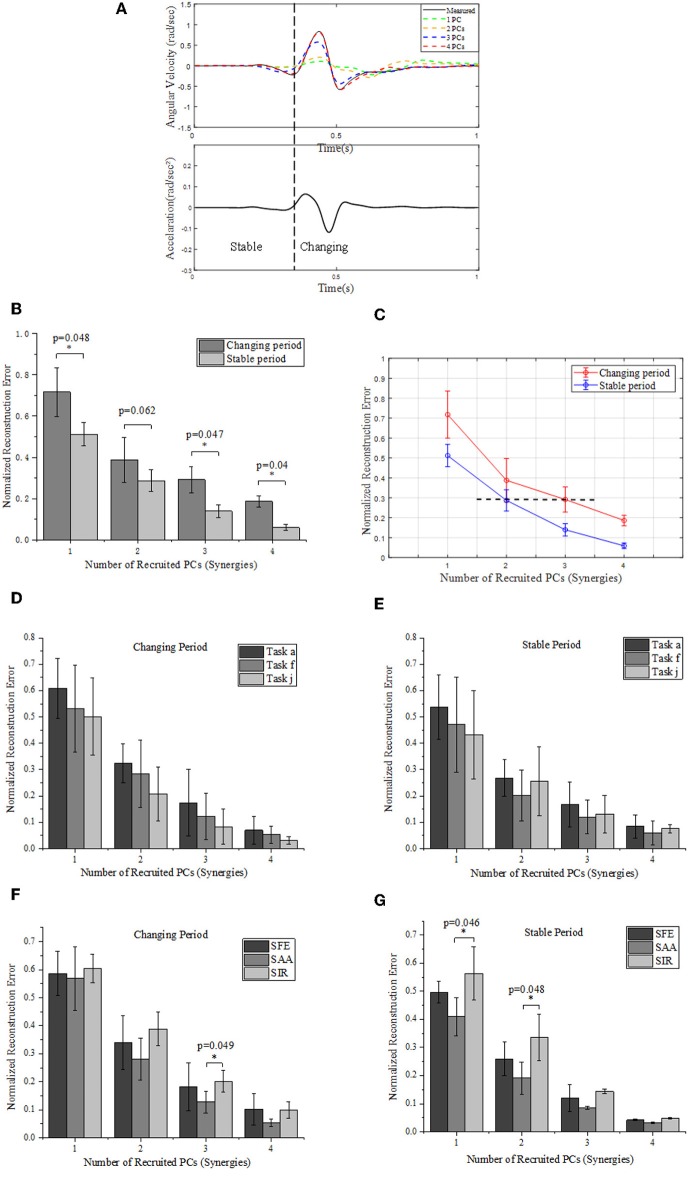
**(A)** Comparison between the measured (solid) and reconstructed (dashed) angular velocity for shoulder abduction of task “a” (Figure 2B) (upper), and the corresponding acceleration of movement (lower). The reconstructed angular velocities presented on the upper plots are calculated based on different numbers of PCs. **(B)** Reconstruction errors of angular velocities within stable and changing periods, respectively. **(C)** Relationships of normalized reconstruction errors against number of PCs (synergies) for angular velocity reconstruction. **(D,E)** Grouped comparison of the impact of synergy number and target position on reconstruction errors in “changing” or “stable” periods. **(F,G)** Grouped comparison of the impact of synergy number and shoulder DOFs on reconstruction errors in “changing” or “stable” periods (*indicates *p* < 0.05).

Figure 7A give an example of the angular velocity reconstruction based on different numbers of PCs (synergies) for shoulder abduction reaching target “a” (Figure 2B). As expected, the reconstructed kinematic profiles for upper limb joints get closer to the actual measurement as the number of synergies augmented. The reconstructed angular velocities with the first 2 synergies (PC1 and PC2 only) can represent similar kinematic trends, such as acceleration/deceleration phases of the real movements, while higher orders of synergies (PC3 and PC4) can supplement and refine the reconstruction to match the true measurements with higher accuracy.

We segment the movements into two specific periods (changing and stable) depending on the value of acceleration: the “changing period” refers to periods when the absolute acceleration value is higher than 20% of the maximum, while the rest period is considered as “stable period” (Figure 7A). We compare the difference of normalized reconstruction errors between the stable and changing period for reconstructed angular velocities (Figure 7B), and significant differences are observed (*p* < 0.05). The normalized errors tends to decrease as the number of PCs (synergies) augmented. In detail, the first 2 synergies yield a normalized reconstruction error of 0.38 in changing period and 0.28 in stable period, while the errors reduce to 0.18 in changing period and 0.05 in stable period when the first 4 PCs (synergies) were involved. Moreover, the normalized errors also vary with movement phases, as the error level for the first 3 PCs in changing period (0.29 ± 0.06) is similar to that for the first 2 PCs in the stable period (0.28 ± 0.05) (see the black dashed line in Figure 7C). In addition, Figure 7C also indicates that the impact of recruited synergy numbers on reconstruction error is greater in the changing period than that in the stable period. When comparing the impact of recruited synergies on reconstruction error among different reaching target positions, it showed similar trend; in other words, either for “changing” or “stable” period, the reconstruction error of upper limb moving decreased with the recruited synergy numbers (see Figures 7D,E). However, as illustrated in Figure 7F, the kinematic synergies showed different roles to reconstruct the moving pattern, and more synergies significantly reduced the reconstruction error in “changing” periods for DOF of shoulder abduction/adduction (SAA). In “stable” period, DOF of shoulder internal/external rotation (SIR) exhibited significant reconstruction error when only the first synergy or the first two synergies were recruited (Figure 7G).

**Figure 8:**
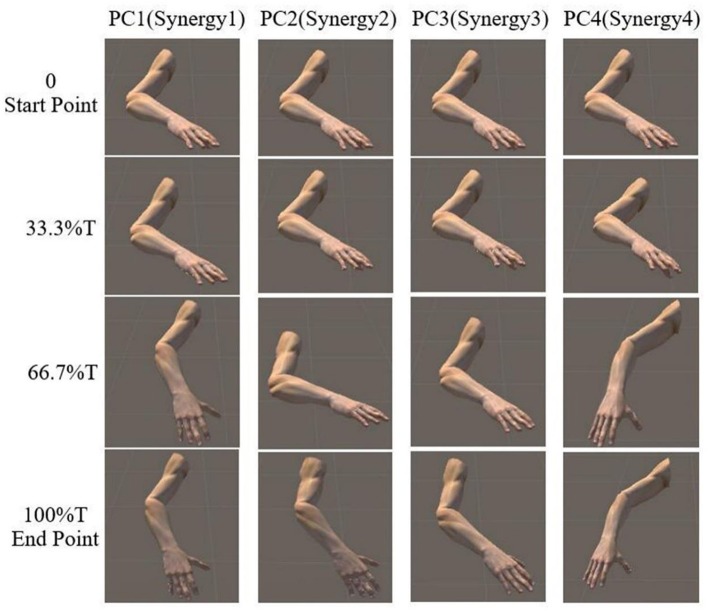
Simulated posture visualization for first four PCs (synergies) at four specific time points of subject 9.

The reaching task conducted by right arm are normalized as an angular trajectory. To simplify the simulation, the reaching movement is divided into 3 equal periods (0–33.3, 33.3–66.6, 66.7–100%T), the angular value for shoulder joints (EFE, SFE, and SAA) and elbow joint (SIR) are obtained through integrating angular velocity with time. Then, the angular values of the DOFs of EFE, SFE, SAA, and SIR are used to drive motor I, motor II, motor III, and motor IV, respectively. An example visualization of simulated postures at four specific time-points for subject 9 are presented in Figure 8. The postures are in accordance with the angular velocity profiles with different synergies as shown in Figure 5.

### Motion Simulation Based on Kinematic Synergies

**Figure 9:**
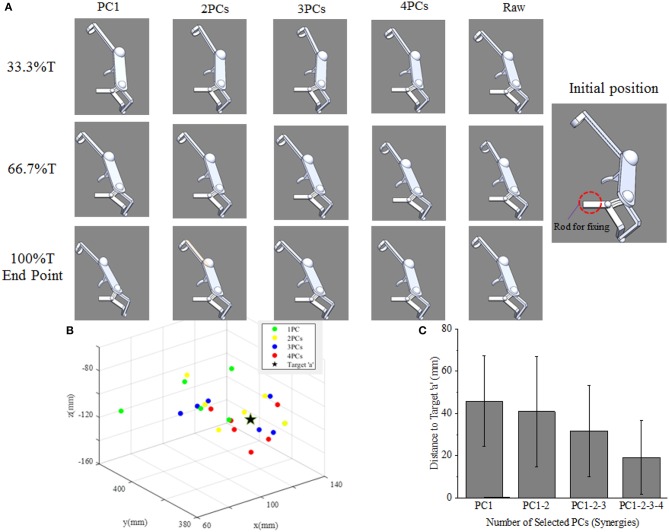
**(A)** The upper limb assistive exoskeleton model driving by the first four PCs (synergies) at three specific time points of subject 5. **(B)** The calculated endpoint positions for subject 5 corresponds to reconstructions based on 1–4 PCs recruitment, with the true position of target “a” (location illustrated by Figure 2B) shown as ⋆. **(C)** Distance errors between calculated and actual target positions for all participants, and its changing trends as the recruited PCs (synergies) augmented.

As shown in Figure 9A, the movement profile of exoskeleton are simulated when applied to assist subject 5 reaching target “a” in SolidWorks. The first four columns demonstrate the postures of assistive exoskeleton when different order of kinematic synergies are employed to control exoskeleton movement at different time point of reaching task. The last column is the movement pattern of assistive exoskeleton when the original kinematic is used to drive the exoskeleton. It can be observed that, with the angular input of shoulder internal/external rotations, shoulder abduction/adduction, shoulder variable for the flexion/extension, and elbow flexion/extension, the assistive exoskeleton moves to target “a” step by step. Figure 9B visually illustrates the endpoint positions of assistive exoskeleton when different number of PCs are involved in D-H model for the subject 5. The distance deviations between simulated reaching points and actual target position are calculated, and the results also show that distance errors of exoskeleton endpoint decrease as the kinematic synergies augmented (Figure 9C).

## Discussion

Motor control optimization is one of the most important issues in assistive arm device design. In our study, the time-varying kinematics synergies of shoulder and elbow joints during upper limb reaching tasks were analyzed using PCA algorithm. Our results showed that the first four principal components can sufficiently represent the dynamical profile of upper limb joint angles, and principal components with different scales exhibited different contributions to the multi-joint motion behaviors. Specifically, the first two synergies (PC1 and PC2) could reflect the direction and motion range of the movement, while the higher order synergies (PC3 and PC4) could smooth the motion trajectory during acceleration period. The motion planning simulation of right arm assistive exoskeleton further confirmed that principal components with different scales played different roles in the motion trajectory and end-point accuracy.

Synergy is an assemble of individual functional units performing motor behaviors in relatively independent DoFs (Turvey, 2007), and can be represented as multi-joint coordination in angular velocity space (Burns et al., 2017). Our results indicated that the first four PCs can be used as surrogates to describe synergistic characteristics since the four PCs were able to explain major proportion of variance. Moreover, we found that the level of variance explained by PCs was significantly reliant on the target position, and the joint coordination patterns were dynamically regulated over time as the number of kinematic synergy (PC) increased (Figures 6A,B). Bockemühl et al. (2010) suggested that the proportion of each principal component was modulated to compensate for the disadvantage of different catching positions. Verrel et al. (2013) proved that the proportion of variance explained by the first three PCs increased when more skilled upper limb movements were performed. In addition, Côté et al. (2002) reported that additional synergy modes were involved when adapting to motion task complexity. Therefore, our results were in accordance with these previous studies, and the correlation coefficient (Figure 6C) further revealed that the kinematic synergy of upper limb was associated with positions of reaching target.

Upper limb movement is conducted through coordination of multiple joints both in time and space (Tomita et al., 2017). As illustrated in Figure 7B, the reconstruction error varied in different motion periods (i.e., changing and stable) defined by angular velocity profiles. This result agreed to a previous finding, in which Ahmad et al. observed that hand motion stability depends on the motion period (Nadzri et al., 2014). Moreover, Mukta et al. reported temporal compensation during the motion starting period under different reach-to-grasp conditions (Mukta et al., 2015). In our study, the error performance in Figure 7C indicated that the reconstruction results could significantly be improved as the number of recruited PCs (synergies) increased. Furthermore, as observed in Figure 7A, the low-order synergies appeared to show the overall change trend of motion, while the high-order synergies reflected the details at the special movement phase, which suggested potential implications for the level assessments of motor function and rehabilitation.

The multi-DoF synergistic pattern of upper limb movements can help to simplify the motion planning of assistive device for human rehabilitation. In fact, synergy has been considered as an effective method to improve motion smoothness of the rehabilitation device as human behavior (Burns et al., 2017). Therefore, implementing the time-varying principal component analysis into the upper limb prosthesis and rehabilitation device was potential to fulfill the requirements. Moreover, recent study (Tsai et al., 2018) investigated joint kinematics regulation of postural system, and confirmed the value of PCs in evaluating the contributions of individual joint. Our results also demonstrated that the low-order synergies or PCs, generally the first two PCs, represented major variance of original kinematics, and could fulfill common movements for rehabilitation devices. On the other hand, to improve the endpoint accuracy of reaching movement, additional high order components (such as the third and fourth PCs) should be involved in. Therefore, specific to daily life activities which generally require precise control, the high-order PCs (synergies) would have advantages on accurate control strategies for assistive device development. Altogether, the simulation results in our study implied the high potential of synergy being implemented for motion planning, and the corresponding precision of endpoints can be improved by synergy augments.

The present work has confirmed that the motion coordination of shoulder and elbow joint is manifested in the coordination of kinematic synergies. With the data from right arm reaching tasks, it can be concluded that different synergies have specific contributions to the upper limb movement, the low-order synergies represented the overall trend of motion patterns, while the high-order synergies described the fine motions at some moving phases. The results of exoskeleton movement simulation further confirmed that kinematic synergies could be used for exoskeleton motion planning, and different principal components contributed to the motion trajectory and end-point accuracy with some specific extent. The findings of this study may provide novel but simplified strategies for the development of rehabilitation and assistive robotic systems approximating the motion pattern of natural upper-limb motor function.

## Data Availability Statement

The raw data supporting the conclusions of this manuscript will be made available by the authors, without undue reservation, to any qualified researcher.

## Ethics Statement

The experimental procedures were conducted in accordance with the Declaration of Helsinki and approved by the Ethical Review Board of Scuola Superiore Sant'Anna.

## Author Contributions

ST collected the data and analyzed the data. AF and WH designed the work. ST, LC, MB, and YL drafted the work. WH, XW, and LB interpreted the data. LH worked on the D-H model.

### Conflict of Interest

The authors declare that the research was conducted in the absence of any commercial or financial relationships that could be construed as a potential conflict of interest.
